# Unprecedented phase transition sequence in the perovskite Li_0.2_Na_0.8_NbO_3_

**DOI:** 10.1107/S2052252517002226

**Published:** 2017-03-08

**Authors:** Charlotte A. L. Dixon, Jason A. McNulty, Steven Huband, Pamela A. Thomas, Philip Lightfoot

**Affiliations:** a School of Chemistry and EaStCHEM, University of St Andrews, St Andrews KY16 9ST, Scotland; b Department of Physics, University of Warwick, Coventry CV4 7AL, England

**Keywords:** perovskite, ferroelectric, powder neutron diffraction

## Abstract

The perovskite Li_0.2_Na_0.8_NbO_3_ is shown, by powder neutron diffraction, to display a unique sequence of phase transitions at elevated temperature. The ambient temperature polar phase (rhombohedral, space group *R*3*c*) transforms *via* a first-order transition to a polar tetragonal phase (space group *P*4_2_ *mc*) in the region 150–300°C; these two phases correspond to Glazer tilt systems *a* ^−^ *a* ^−^ *a* ^−^ and *a* ^+^ *a* ^+^ *c* ^−^, respectively. At 500°C a ferroelectric–paraelectric transition takes place from *P*4_2_ *mc* to *P*4_2_/*nmc*, retaining the *a* ^+^ *a* ^+^ *c* ^−^ tilt. Transformation to a single-tilt system, *a* ^0^ *a* ^0^ *c* ^+^ (space group *P*4/*mbm*), occurs at 750°C, with the final transition to the aristotype cubic phase at 850°C. The *P*4_2_ *mc* and *P*4_2_/*nmc* phases have each been seen only once and twice each, respectively, in perovskite crystallography, in each case in compositions prepared at high pressure.

## Abstract 2

We demonstrate the existence of a novel phase transition sequence in the perovskite Li_0.2_Na_0.8_NbO_3_ at elevated temperature. The rare Glazer tilt system, *a* ^+^ *a* ^+^ *c* ^−^, is observed in both polar and centrosymmetric polymorphs.

## 1. Introduction   

Perovskites continue to fascinate solid-state scientists, both for their chemical and structural diversity and for their ever-growing list of physical and chemical properties and applications (Chakhmouradian & Woodward, 2014 ▸; Jeon *et al.*, 2014 ▸). Considerable effort in the last few years has focused on alkali metal niobate perovskites for their promising piezoelectric and electro-optic properties (Jo *et al.*, 2009 ▸; Saito *et al.*, 2004 ▸). Within this family, the Li_*x*_Na_1 − *x*_NbO_3_ (hereafter abbreviated LNN-*X*, where *X* represents the % Li composition) system has been shown to display some curious features at ambient temperature (Peel *et al.*, 2013 ▸). Detailed crystallographic characterization of this system at elevated temperatures has only been reported for one specific composition, LNN-12 (Mishra *et al.*, 2015 ▸). As part of a more comprehensive study of the high-temperature phase diagram of LNN-*X* we now report a detailed study of LNN-20 by high-resolution powder neutron diffraction. This reveals some unique structural behaviour, including two very rare structural variants of perovskite, based on an unusual combination of tilting of NbO_6_ octahedra.

## 2. Experimental   

### 2.1. Synthesis   

LNN-20 was prepared by traditional ceramic methods. Stoichiometric amounts of Na_2_CO_3_ (99.9% Sigma-Aldrich), Li_2_CO_3_ (99.9% Sigma-Aldrich) and Nb_2_O_5_ (99.9% Alfa Aesar) were dried at 100°C for 24 h, ground for a period of 30 min and pressed into pellets of approximately 10 mm diameter and 5 mm thickness. The pellet was then annealed at 1000°C for a period of 24 h, before being cooled at a rate of 10° min^−1^. We assume no deviation of alkali metal content due to volatility, and a fixed Li/Na and (Li,Na)/Nb ratio in the subsequent analysis.

### 2.2. Powder diffraction   

Sample purity was analysed by powder X-ray diffraction using a Rigaku miniflex 600 X-ray diffractometer using Cu *K*α_1,2_ (λ = 1.54056, 1.54439 Å) radiation. Powder neutron diffraction (PND) data were collected on the HRPD instrument at the ISIS facility at selected temperatures between 20 and 900°C. The sample was contained in a cylindrical vanadium can. For data analysis, two detector banks, centred at ∼ 168° and 90°, and covering *d*-spacing ranges of 0.7 < *d* < 2.6 Å and 0.9 < *d* < 4.0 Å, respectively, were used. Data were analysed by the Rietveld method, using the *GSAS*/*EXPGUI* package (Larson & Von Dreele, 1994 ▸; Toby, 2001 ▸), and interpretation of the results was assisted using symmetry mode analysis *via* the *ISODISTORT* suite (Campbell *et al.*, 2006 ▸). A consistent refinement strategy was used in each case, including 3, 2, 18 and 6 parameters to model instrumental variables, scale factors, background and peak-shapes for each dataset, together with appropriate lattice parameters, atomic coordinates (Li and Na constrained to be equal) and atomic displacement parameters (Li and Na constrained to be equal, and some additional constraints, detailed later).

### 2.3. Second-harmonic generation (SHG)   

A polycrystalline powder sample of LNN-20 was contained in glass cells capable of withstanding a high-temperature study. Calibration of the furnace was then performed, followed by null checks, before the sample was placed in a small furnace consisting of an aperture normal to the orientation of the sample. Through these apertures an intense beam (100 mJ, 100 ms pulse) was exposed to the sample from a Q-switched Nd:YAG laser (λ = 1064 nm) following the setup of Kurtz & Perry (1968 ▸). Filtration with a green filter of the scattered radiation was carried out to remove traces of the fundamental beam whilst the second harmonic signal (if any was present) was measured using a photomultiplier (PMT) and subsequently displayed on an oscilloscope as signal *versus* time. A beam splitter and photodiode were utilized to monitor the fundamental beam, allowing for the necessary corrections to variations in the laser output. The intensity of the second harmonic was then normalized by dividing by the square of the intensity of the incident beam (Kurtz & Perry, 1968 ▸). Repeat measurements as a function of temperature were made to remove the effects of any residual strain from the sample preparation and to ensure the reproducibility of the results.

### 2.4. Dielectric measurements   

Pellets were electroded with Ag paste (*RS* components) or sputtered Au. Dielectric measurements were performed with a Wayne Kerr 6500B impedance analyser with the sample mounted in a tube furnace. Capacitance and loss data were recorded in the frequency range 100 Hz–10 MHz on heating and cooling at a rate of 2 K min^−1^ over the temperature range ambient to approximately 600°C.

## 3. Results and discussion   

### 3.1. Thermal evolution of the crystal structure   

#### 3.1.1. Room temperature to 500°C   

At ambient temperature the PND data can be successfully modelled using a majority perovskite phase of space-group symmetry *R*3*c*, in agreement with our earlier work (Peel *et al.*, 2013 ▸). There is a minor additional rhombohedral phase present all the way to 900°C; this phase corresponds to a LiNbO_3_-like material, essentially isostructural to the majority perovskite phase, but of composition close to LiNbO_3_. The co-existence of two distinct rhombohedral phases has been noted in our previous work (Peel *et al.*, 2013 ▸), with the Li-rich phase being labelled ‘Li-*R*3*c*’ in that work. Two phase refinements suggest a phase fraction, constant *versus T*, of only ∼ 3% for the Li-*R*3*c* phase. Thus for simplicity, and to allow more valid comparisons of the various models to be discussed for the majority perovskite phase, we have chosen not to incorporate this minor phase into all of the refinements presented here. It is most clearly seen at the highest temperatures, and is highlighted in a two-phase refinement of the data at 900°C (see Fig. S3 of the supporting information). All further discussions omit reference to the minor Li-*R*3*c* phase.

The majority rhombohedral phase, which corresponds to the Glazer tilt system 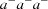, but also includes an additional polar distortion, may be considered isostructural with the low-temperature form of NaNbO_3_ (and as a less distorted version of the stable phase of LiNbO_3_). On warming to 150°C a second phase is seen to appear, which becomes the only phase present by 300°C (these two phases co-exist at temperatures of 150, 200 and 250°C, with phase fractions of ∼ 74/26, 20/80 and 7/93%, respectively, which provides evidence of a first-order phase transition).

Careful Rietveld analysis is required to ascertain the exact nature of the higher-temperature phase. Perovskite crystallography is often dominated by the presence of characteristic ‘octahedral tilt’ distortions: in simple cases (*i.e.* for a 2 × 2 × 2 array of corner-linked octahedra) there are options of ‘in-phase’ or ‘out-of-phase’ tilts relative to each of the three axes of the parent cubic phase. The possible combinations of these tilts were first classified by Glazer (1972 ▸) and subsequently re-assessed using group-theoretical methods to produce 15 unique possibilities (Howard & Stokes, 1998 ▸). Thus, for example, the Glazer symbol 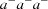 signifies equal out-of-phase tilts around all three axes of the parent cubic phase. The simplest and least ambiguous means of determining which tilt system is present is to identify characteristic peaks in the diffraction pattern which are due to either in-phase or out-of-phase tilts. These independent sets of peaks may be designated by the labels *M* and *R*, or more specifically *M_3_* ^+^ (in-phase tilt) and *R_4_^+^* (out-of-phase tilt).

**Figure 1:**
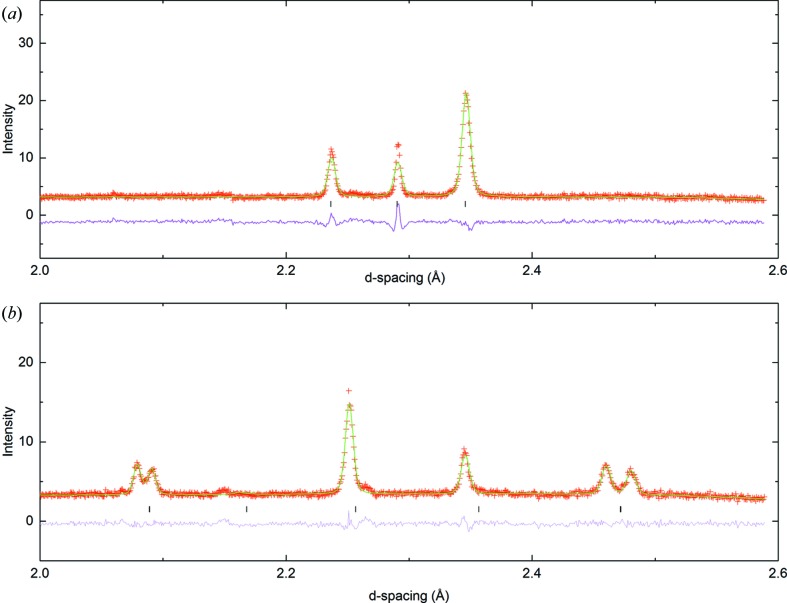
Portions of Rietveld refinement carried out on PND data at (*a*) 100°C and (*b*) 300°C. In (*a*) the peaks in the region 2.2–2.3 Å arise from the subcell; the peak near 2.35 Å in both (*a*) and (*b*) is due to the *R*_4_^+^ tilt mode. The additional peaks (‘doublets’ near *d* = 2.09 and 2.48 Å) in (*b*) are due to the *M*_3_^+^ tilt mode. The small peak near *d* = 2.14 Å is from the vanadium sample holder and the ‘shoulder’ near *d* = 2.27 Å is due to the minority Li-*R*3*c* phase.

Key portions of the diffraction patterns of LNN-20 at 100°C and 300°C are shown in Fig. 1 ▸. It can be readily seen that superlattice peaks (due predominantly to octahedral tilting) are located at *R* positions only in the former, but at both *R* and *M* positions in the latter; in other words, in addition to out-of-phase tilts, the higher-temperature phase contains in-phase tilts. The key question is ‘what are the directions of the constituent tilts relative to the cubic parent phase’?

The analysis of Howard & Stokes (1998 ▸) suggests four possible structural models comprising both types of tilts, designated 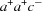, 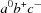, 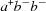 and 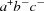. The first three of these were each tested against the PND data at 300°C [the latter has unnecessarily low (monoclinic) symmetry and is discarded]. The first two models are described in space groups *P*4_2_/*nmc* and *Cmcm*, respectively, with unit cells of approximate metrics 2 × 2 × 2 *a*_p_, where *a*_p_ represents the aristotype cubic perovskite unit-cell parameter. Both these models give good quality fits, whereas the 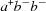 option (space group *Pnma*, which corresponds to the most common perovskite tilt system, with unit-cell metrics approximately 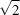 × 2 × 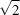 *a*_p_), does not adequately describe the observed peak splittings.

**Figure 2:**
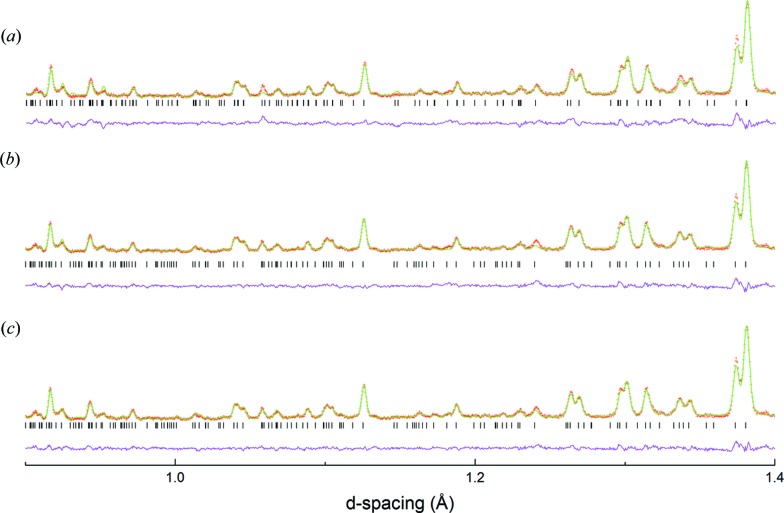
Portion of Rietveld refinement (PND) of LNN-20 at 300°C modelled in (*a*) *Cmcm*; (*b*) *P*4_2_/*nmc* and (*c*) *P*4_2_ *mc* space groups.

Further details of the two most promising models, *P*4_2_/*nmc* and *Cmcm*, are given in Table 1 ▸, together with several models representing non-centrosymmetric subgroups of *P*4_2_/*nmc* (see later). Both centrosymmetric structural models have the same number of variable atomic parameters (coordinates plus isotropic displacement parameters) which makes direct numerical comparison of fit quality straightforward. In addition to the significantly improved fit (χ^2^), the 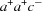 (*P*4_2_/*nmc*) model can be seen graphically to be significantly better (Fig. 2 ▸). Moreover the atomic displacement parameters for the O atoms show much more uniform behaviour [*U*_iso_ (Å^2^) values for the three distinct O atoms in the *P*4_2_/*nmc* and *Cmcm* models, respectively are 0.0152 (7), 0.0136 (7), 0.0179 (4) and 0.0137 (6), −0.0028 (6), 0.0348 (10)] – a clear indication that the octahedral tilt behaviour is modelled correctly in the 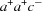 model and incorrectly for 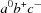.

**Figure 3:**
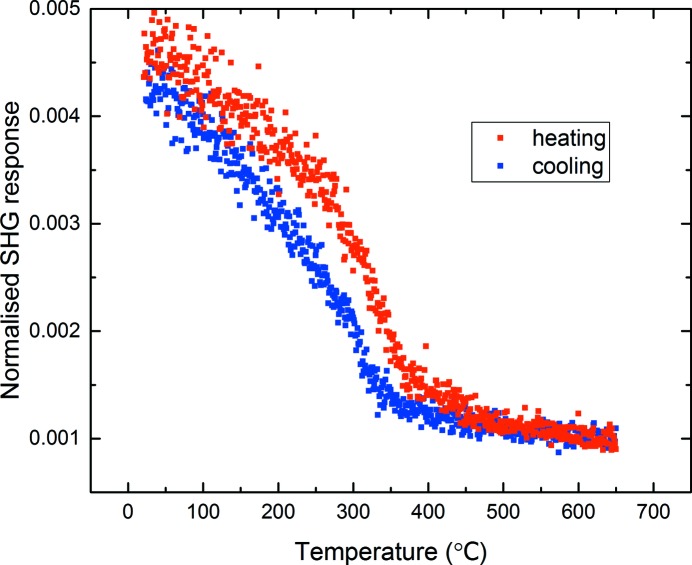
SHG data generated from a sample of LNN-20 showing clear SHG response to at least 450°C on heating.

The above analysis confirms the tilt system of the phase present at 300°C to be 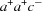. However, this assumes the phase to be centrosymmetric. Second harmonic generation offers a sensitive powder technique with the ability to establish the centrosymmetric or non-centrosymmetric nature of a crystalline phase, independently of diffraction data. We therefore carried out second-harmonic generation (SHG) measurements as a function of temperature, as shown in Fig. 3 ▸.

**Figure 4:**
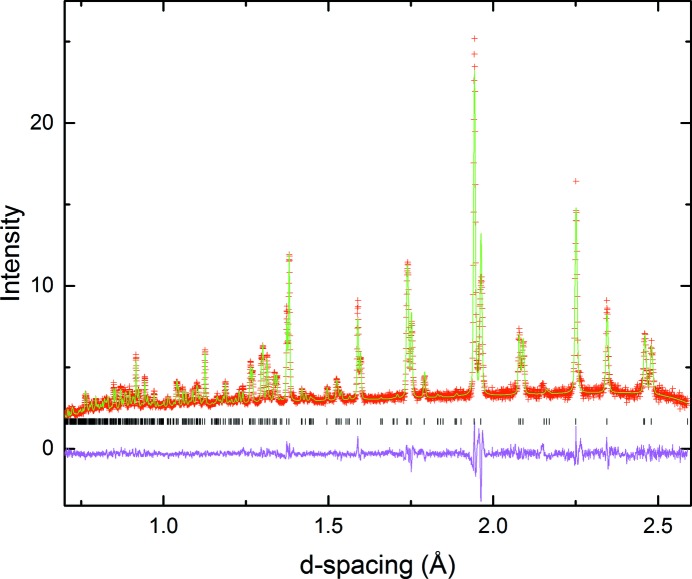
Portion of Rietveld refinement (PND) of LNN-20 at 300°C using the *P*4_2_ *mc* model.

This experiment clearly shows a significant SHG signal to at least 450°C, thus supporting the presence of a non-centrosymmetric phase. Hence, the simplest non-centrosymmetric subgroups of *P*4_2_/*nmc* were considered, and these models are presented in Table 1 ▸. All four potential models were treated similarly with the same set of ‘standard’ refined parameters, as described in §2.1. Atomic coordinates and displacement parameters were then refined for each of the models with displacement parameters constrained for those crystallographic sites that were ‘split’ in two upon distortion to the lower symmetry phase. It can be seen that the *P*4_2_ *mc* model provides the best description of the phase present in the region 300 < *T* < 500°C, consistent with both the observed tilt system and the presence of SHG signal. We note that this is the only *polar* subgroup; the other three subgroups tested are non-centrosymmetric but *not* polar. The final, refined model for space group *P*4_2_ *mc* at 300°C is presented in Table 2 ▸, and the corresponding Rietveld fit in Fig. 4 ▸. One further point here is worthy of comment: the *U*_iso_ values of the *A*-site cations may suggest a possible partial ordering of Li/Na over these sites (Li has a negative scattering length); we tested this, but no clear-cut ordering pattern or significant lowering of agreement parameters was observed.

#### 3.1.2. 500 < *T* < 850°C   

**Figure 5:**
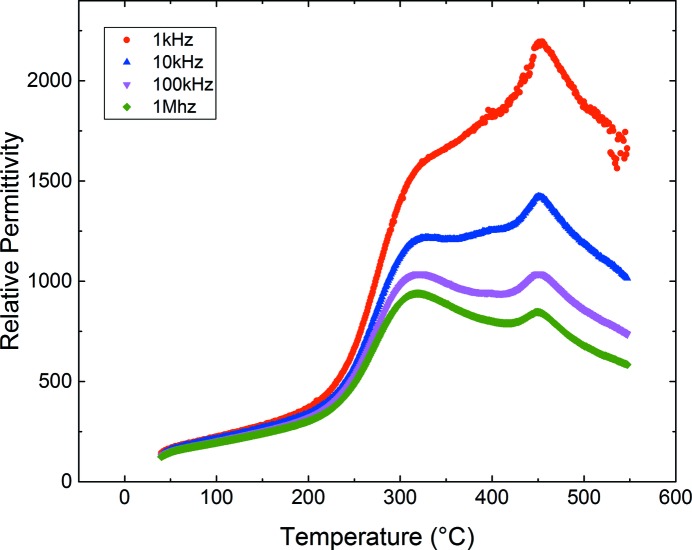
Relative permittivity data for LNN-20 at selected frequencies, obtained on cooling.

In addition to the SHG signal (Fig. 3 ▸), which supports the presence of a non-centrosymmetric LNN-20 phase to at least 450°C, dielectric data support an additional structural event in the region of 450–500°C (Fig. 5 ▸).

**Figure 6:**
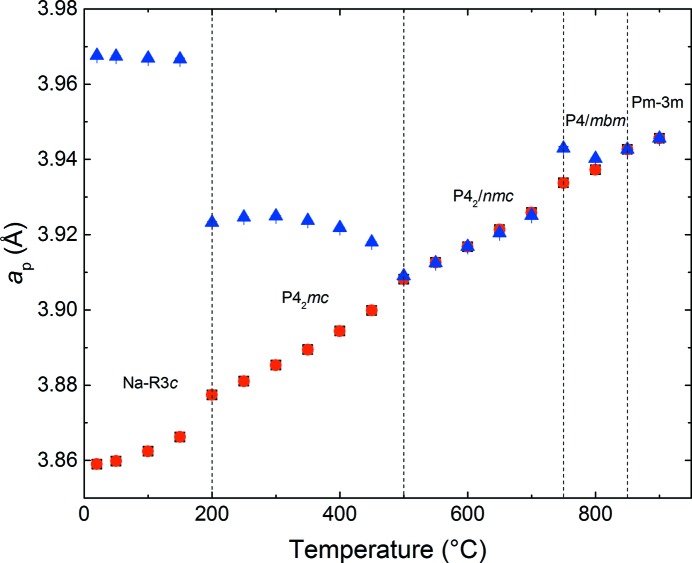
Normalized lattice parameters *versus T*, obtained from Rietveld refinement of PND data. For the tetragonal phases, the *a* lattice parameter is represented by red circles and *c* by blue triangles. In regions where phase co-existence occurs, only the majority phase present at each temperature is shown.

Further corroborating evidence of a phase transition in this region could be seen from the PND data, with a sharp merging of several peaks when the temperature was elevated from 450°C to 500°C. For completeness the data at 500°C were modelled in the *P*4_2_ *mc* space group and whilst the χ^2^ was lower for *P*4_2_ *mc* over *P*4_2_/*nmc* (2.75 and 3.17, respectively) this was most likely an artefact due to the increased degrees of freedom in the lower symmetry space group; indeed it was found that the model was not stable upon full, unrestrained refinement. Key superlattice peaks corresponding to both *M*-point and *R*-point tilts are still observed above 500°C, however. In light of this, the models for each of the standard 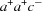, 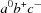 and 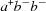 tilt systems, previously discounted for the phase at 300°C, were trialled as fits to this new (presumed centrosymmetric) phase at 500°C. Of these three tilt systems the fit to the *P*4_2_/*nmc* model was clearly the most satisfactory at this temperature (χ^2^ value for *P*4_2_/*nmc* = 3.175, *Pnma* = 4.045, *Cmcm* = 4.442). Evolution of the lattice parameters *versus* temperature, based on the *P*4_2_ *mc* (250–450°C), *P*4_2_/*nmc* (500–700°C) and subsequent higher temperature phases is shown in Fig. 6 ▸. We note, in particular, the marked tetragonal distortion in the *P*4_2_ *mc* phase field, related to the polar cation displacements along *c*. This distortion disappears on entering the pseudo-cubic *P*4_2_/*nmc* phase, but an enhanced tetragonality reappears at 750°C, with the gradual emergence of a new phase. Finer temperature increments would be necessary to establish a more precise point for the *P*4_2_ *mc* to *P*4_2_/*nmc* phase transition, but this clearly occurs in the range 450°C to 500°C, consistent with the SHG, dielectric data and PND.

As the centrosymmetric *P*4_2_/*nmc* space group remained the best fit to the diffraction data in the region 500 ≤ *T* ≤ 600°C, a single phase refinement using this model was carried out over these temperatures. Upon reaching 650°C further structural changes were indicated in the diffraction data, with a developing asymmetry of several peaks. This new phase was seen to ‘grow in’ as the temperature was increased to 800°C where complete loss of the superlattice peaks attributed to the *R*_4_^+^ out-of-phase tilt mode occurs, suggesting complete conversion to the emergent phase. For a centrosymmetric perovskite with only *M*_3_^+^ (in-phase) tilts, the analysis of Howard & Stokes (1998 ▸) suggests three possible tilt systems; 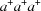 (space group 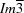), 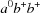 (*I*4/*mmm*) and 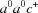 (*P*4/*mbm*). Of these, the 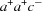 to 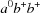 transition is expected to be second order; clearly this is not the case here. The other two options are required to be first order, compatible with the phase co-existence observed here. The cubic 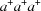 option can easily be discounted, thus leaving the tilt system 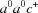 (*P*4/*mbm*) as the only viable option.

**Figure 7:**
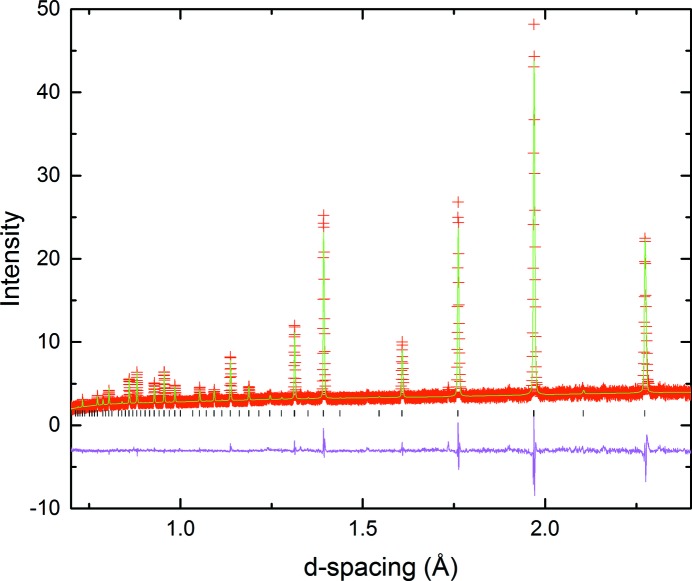
Portion of Rietveld refinement (PND) of LNN-20 at 800°C using the *P*4/*mbm* model.

The tetragonal model *P*4/*mbm* with 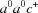 tilt system was indeed confirmed to provide the best fit. A two phase refinement of *P*4_2_/*nmc* and *P*4/*mbm* models was carried out for 650 ≤ *T* ≤ 750°C, with a single phase refinement in the *P*4/*mbm* space group for the 800°C diffraction data (Fig. 7 ▸). Elevation of the temperature to 850°C resulted in the loss of all remaining superlattice peaks, signalling the disappearance of the in-phase tilt along the *c*-axis. All subsequent data were therefore fitted to the aristotype cubic space group 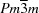.

### 3.2. Discussion   

The phase transition sequence reported here (*R*3*c* – *P*4_2_ *mc* – *P*4_2_/*nmc* – *P*4/*mbm* – 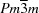) is unique in perov­skite crystallography. Moreover, the unusual structural variants displaying the 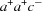 tilt system (space groups *P*4_2_/*nmc* and *P*4_2_ *mc*) present throughout the intermediate temperature range studied here have only been previously reported twice and once each, respectively. The general trends in octahedral tilt preferences *versus* temperature (*i.e.* from ‘in-phase’ tilts only at high temperature to ‘out-of-phase’ tilts only at low temperature) mirror those in NaNbO_3_ itself (Megaw, 1974 ▸; Peel *et al.*, 2012 ▸), although the specific details are quite different in the two cases, with NaNbO_3_ showing more complex, longer-range tilt sequences. Such longer-range superlattices can be definitively ruled out for LNN-20 from the present data.

**Figure 8:**
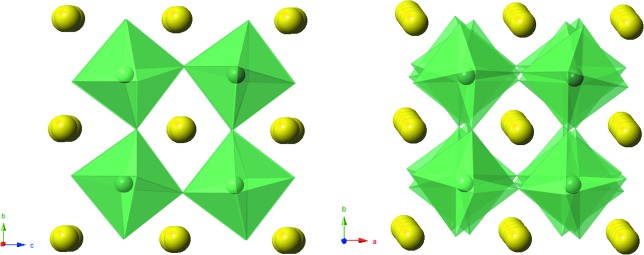
Crystal structure of the *P*4_2_ *mc* phase at 300°C showing (*a*) the view down the *a*-axis highlighting in-phase tilting in this direction, and off-centring of Nb atoms along the polar *c*-axis and (*b*) the out-of-phase tilt along the *c*-axis.

**Figure 9:**
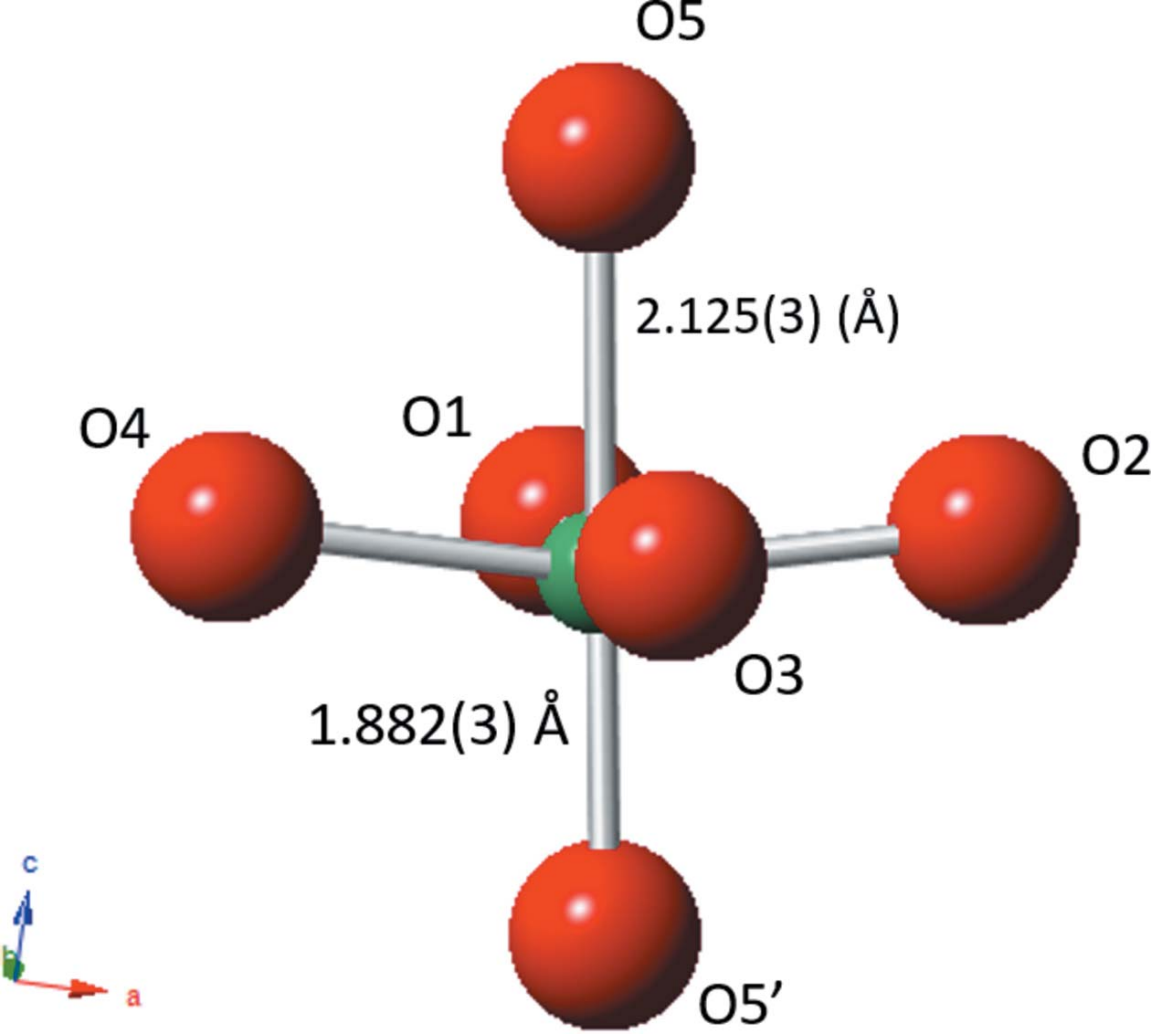
Schematic of NbO_6_ octahedron in the *P*4_2_ *mc* phase at 300°C highlighting the polar Nb displacement (difference between Nb—O5 and Nb—O5′ bond lengths ∼ 0.243 Å). The symmetry operators acting upon the Nb—O5 and Nb—O5′ bonds are 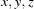 and 
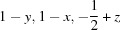
, respectively.

**Figure 10:**
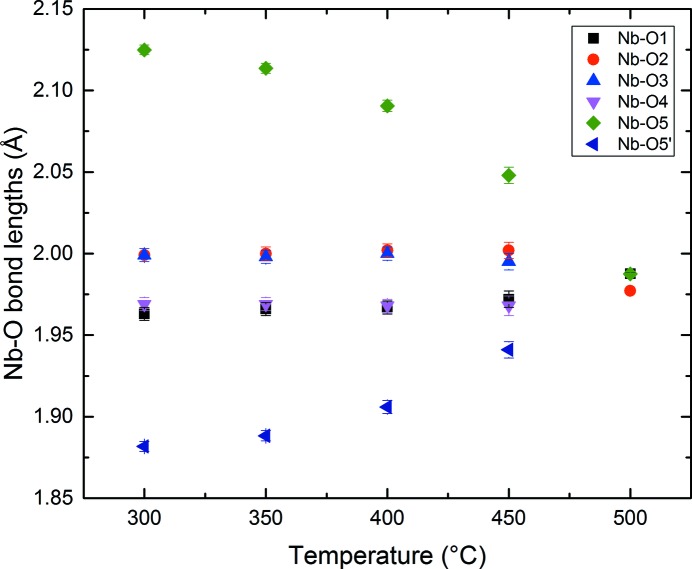
Thermal evolution of Nb—O bond lengths over the region 300 < *T* < 500°C, showing a decrease in the off-centring of the Nb atom within the NbO_6_ octahedron as the *P*4_2_ *mc* – *P*4_2_/*nmc* phase transition is approached.

**Figure 11:**
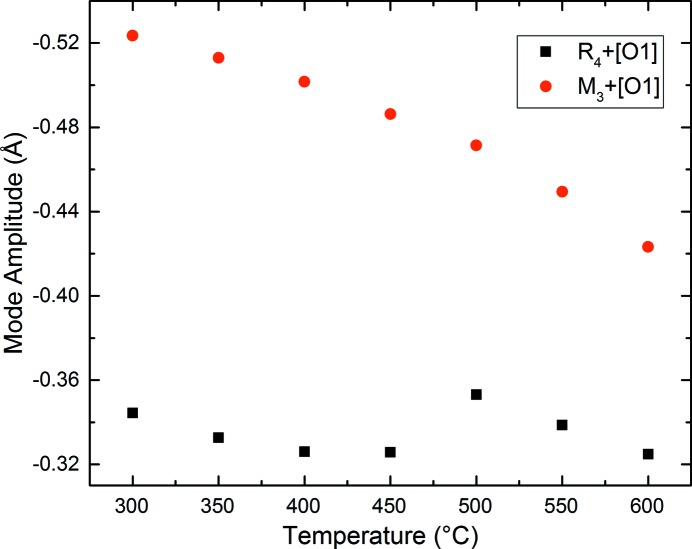
Thermal evolution of the two tilt modes throughout the *P*4_2_ *mc* – *P*4_2_/*nmc* phase field. Note the discontinuous evolution of the *R*_4_^+^ mode.

The crystal structure of the ambient temperature, *R*3*c*, phase has been discussed previously (Peel *et al.*, 2013 ▸). The crystal structure of the *P*4_2_ *mc* phase at 300°C is shown in Fig. 8 ▸. Corresponding bond lengths and selected bond angles are given in Table 3 ▸. Fig. 9 ▸(*a*) shows the distortion of the NbO_6_ octahedron. The off-centre displacement of the Nb atom, along the polar *c*-axis, is evident, and resembles that in much simpler tetragonal ferroelectric perovskites (without octahedral tilting) such as the ambient-temperature phase of BaTiO_3_. In contrast to this difference of ∼ 0.243 Å in axial Nb—O bond lengths, the corresponding *ab*-plane bond lengths are roughly equal. The thermal evolution of these Nb—O bonds is shown in Fig. 10 ▸, and this clearly shows the centralization of the Nb atoms within its octahedral site as the *P*4_2_ *mc* – *P*4_2_/*nmc* transition is approached. The variation in the two octahedral tilt mode, *M*_3_^+^ and *R*_4_^+^, *versus* temperature across the *P*4_2_ *mc* – *P*4_2_/*nmc* phase region is shown in Fig. 11 ▸. The *M*_3_^+^ mode amplitude shows a uniform reduction with increasing temperature, whereas the *R*_4_^+^ mode exhibits a step change at the 500°C transition, presumably in order to accommodate the transition to the centrosymmetric state.

As suggested earlier, the 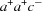 Glazer tilt system has only been observed very rarely: twice in its aristoype form, space group *P*4_2_/*nmc* (Leinenweber & Parise, 1995 ▸; Shimura *et al.*, 2017 ▸), once in the polar form, *P*4_2_ *mc*, also observed here (Aimi *et al.*, 2014 ▸), and additionally in a *B*-site ordered variant, space group *P*4_2_/*n* (Solana-Madruga *et al.*, 2016 ▸). Each of these earlier reports relate to compounds prepared at high pressure, *viz.* CaFeTi_2_O_6_ (Leinenweber & Parise, 1995 ▸), MnLnMnSbO_6_ (Solana-Madruga *et al.*, 2016 ▸), CaMnTi_2_O_6_ (Aimi *et al.*, 2014 ▸) and LnMn(Ga_0.5_Ti_0.5_)_2_O_6_ (Shimura *et al.*, 2017 ▸). Each of these high-pressure-prepared phases incorporate unusually small cations (Fe^2+^ or Mn^2+^) at the *A*-site, and consequently exhibit complete *A*-site cation ordering across geometrically distinct sites. In particular the Fe^2+^ or Mn^2+^ cations in CaFeTi_2_O_6_ and CaMnTi_2_O_6_ occupy two distinct sites of approximately tetrahedral and square-planar geometry, each having four significantly shortened *A*—O contacts. Although it is tempting to suggest that such a structure might be chosen in the present case in order to permit a degree of Li/Na ordering, there is insufficient evidence from the present data to support this possibility. Aimi *et al.* (2014 ▸) reported ferroelectric hysteresis at room temperature in CaMnTi_2_O_6_, and a phase transition to the paraelectric *P*4_2_/*nmc* phase at 630 K. Unusually they show that this transition has a partial order–disorder nature, with the square-planar Mn site (only) displaying disorder in the *P*4_2_/*nmc* phase. Again, no evidence for such an occurrence can be definitively gleaned from the present study. The off-centre displacement of the *B*-site cation (Ti^4+^) in CaMnTi_2_O_6_ has a similar magnitude to that reported for Nb^5+^ here (Fig. 9 ▸). However, the geometries of the *A*-site cations are quite different between LNN-20 and CaMnTi_2_O_6_. Rather than having well defined four-coordinate geometries the *A*-sites in LNN-20 have a less regular geometry, as shown in Figs. S5 and S6.

The third tetragonal polymorph observed in the present study (the *P*4/*mbm* phase) is observed in the high-temperature phase diagram of NaNbO_3_, and also occurs in the variable-temperature phase diagram of several other perovskites, for example, NaMgF_3_ (Knight *et al.*, 2015 ▸) and CsSnI_3_ (Yamada *et al.*, 1991 ▸). On transforming to the cubic phase at 850°C, the remaining in-phase tilts are lost, although some evidence for residual localized octahedral tilting can be inferred from the anisotropic nature of the O atom displacement parameters [*U* ^11^ = 0.0094 (5), *U* ^33^ = 0.0677 (4) Å^2^].

## 4. Conclusions   

The thermal evolution of the phase behaviour and crystal structure of the perovskite ferroelectric Li_0.2_Na_0.8_NbO_3_ (LNN-20) has been characterized using powder neutron diffraction, supported by second-harmonic generation and dielectric measurements. Persistence of a non-centrosymmetric perovskite phase was confirmed up to at least 500°C. The phase progression is determined as *R*3*c* – *P*4_2_ *mc* – *P*4_2_/*nmc* – *P*4/*mbm* – 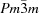 on increasing temperature from ambient to 900°C. This phase transition sequence demonstrates the identification of two rare phases with regard to the perovskite structure: both the polar *P*4_2_ *mc* and centrosymmetric *P*4_2_/*nmc* phases have the Glazer tilt system 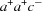, and each structure type has been reported only once or twice previously in a perovskite. The ultimate reasons for Li_0.2_Na_0.8_NbO_3_ to adopt such an unusual sequence of phases remains to be determined.

## Supplementary Material

Crystal structure: contains datablock(s) LNN20R3C_publ, LNN20R3C_overall, LNN20R3C_phase_1, LNN20R3C_phase_2, LNN20R3C_p_01, LNN20R3C_p_02. DOI: 10.1107/S2052252517002226/fc5017sup1.cif

Crystal structure: contains datablock(s) P42NMC_publ, P42NMC_overall, P42NMC_phase_1, P42NMC_phase_2, P42NMC_p_01, P42NMC_p_02. DOI: 10.1107/S2052252517002226/fc5017sup2.cif

Crystal structure: contains datablock(s) LNN20_P4MBM_800C_publ, LNN20_P4MBM_800C_overall, LNN20_P4MBM_800C_phase_1, LNN20_P4MBM_800C_phase_2, LNN20_P4MBM_800C_p_01, LNN20_P4MBM_800C_p_02. DOI: 10.1107/S2052252517002226/fc5017sup3.cif

Crystal structure: contains datablock(s) LNN20_900C_CUBIC_publ, LNN20_900C_CUBIC_overall, LNN20_900C_CUBIC_phase_1, LNN20_900C_CUBIC_phase_2, LNN20_900C_CUBIC_p_01, LNN20_900C_CUBIC_p_02. DOI: 10.1107/S2052252517002226/fc5017sup4.cif

Crystal structure: contains datablock(s) LNN20_P42MC_300C_publ, LNN20_P42MC_300C_overall, LNN20_P42MC_300C_phase_1, LNN20_P42MC_300C_phase_2, LNN20_P42MC_300C_p_01, LNN20_P42MC_300C_p_02. DOI: 10.1107/S2052252517002226/fc5017sup5.cif

Raw powder neutron diffraction data URL: https://doi.org/10.17630/c97a6cc7-05e8-4f3a-b881-0ee334dee69d

## Figures and Tables

**Table 1:** Comparison of the respective Rietveld refinements for the two chosen centrosymmetric models proposed for the phase at 300°C, together with the feasible non-centrosymmetric subgroups of *P*4_2_/*nmc* *N*_ref_ defines the total number of variable parameters refined, *N_xyz_* is the number of variable atomic coordinates and 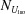 the number of isotropic atomic displacement parameters (ADPs) refined. Occupancies of the *A*-sites are fixed at 80% Na/20% Li in each case, with Li/Na at each particular site having the same ADP. For the non-centrosymmetric models, O atom ADPs were constrained in pairs according to the parent phase, as appropriate

Space group	χ^2^	*N*_ref_	*N*_*xyz*_	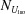
*P*4_2_ *mc*	3.513	55	17	7
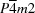	4.263	55	17	7
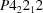	4.428	50	12	7
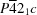	4.582	51	13	7
*P*4_2_/*nmc*	4.553	44	7	7
*Cmcm*	6.692	44	7	7

**Table 2:** Crystallographic data for LNN-20 at 300°C modelled in the *P*4_2_ *mc* space group [*a* = 7.7706 (2), *c* = 7.8498 (3) Å]

Atom	Wycoff position	*x*	*y*	*z*	100 × *U*_iso_ (Å^2^)
Na1†	2*c*	0	0.5	0.726 (3)	4.1 (3)
Na2	2*c*	0	0.5	0.258 (6)	4.1 (3)
Na3	2*b*	0.5	0.5	0.253 (3)	0.2 (4)
Na4	2*a*	0	0	0.236 (2)	−0.7 (3)
Nb1	8*f*	0.2489 (5)	0.7493 (5)	0	0.48 (2)
O1	4*e*	0.2281 (8)	0.5	0.4843 (7)	1.39 (9)
O2	4*e*	0.2781 (8)	0.5	0.0552 (7)	0.75 (7)
O3	4*d*	0.2864 (8)	0	0.5538 (8)	0.75 (7)
O4	4*d*	0.2115 (8)	0	0.0026 (6)	1.39 (9)
O5	8*f*	0.2858 (10)	0.7874 (11)	0.2655 (4)	1.52 (4)

† Positions Na1–Na4 have fixed occupancy Na_0.8_Li_0.2_; the derived *U*_iso_ values (especially the unphysical value for Na4) illustrate the possibility of a slight Na/Li ordering.

**Table 3:** Bond lengths and selected bond angles of the *P*4_2_ *mc* phase at 300°C, Na—O bond lengths over 2.7 Å are not reported

Na—O	Bond length (Å)	Nb—O	Bond length (Å)	Nb—O—Nb	Bond angle (°)
Na1—O1 × 2	2.597 (17)	Nb—O1	1.963 (4)	Nb—O1—Nb	167.8 (4)
Na1—O5 × 4	2.365 (3)	Nb—O2	1.999 (4)	Nb—O2—Nb	150.8 (4)
Na2—O1 × 2	2.51 (3)	Nb—O3	1.999 (4)	Nb—O3—Nb	151.1 (4)
Na2—O2 × 2	2.69 (3)	Nb—O4	1.969 (4)	Nb—O4—Nb	163.3 (4)
Na2—O3 × 2	2.31 (3)	Nb—O5	2.125 (3)	Nb—O5—Nb	156.77 (10)
Na3—O2 × 2	2.322 (17)	Nb—O5’	1.882 (3)		
Na4—O3 × 2	2.644 (12)				
Na4—O4 × 2	2.460 (15)				
Na4—O4 × 2	2.664 (15)				
